# Timing of Assessment of Humoral and Cell-Mediated Immunity after Influenza Vaccination

**DOI:** 10.3390/vaccines12060584

**Published:** 2024-05-27

**Authors:** Naruhito Otani, Kazuhiko Nakajima, Kumiko Yamada, Kaori Ishikawa, Kaoru Ichiki, Takashi Ueda, Yoshio Takesue, Takuma Yamamoto, Satoshi Higasa, Susumu Tanimura, Yuta Inai, Toshiomi Okuno

**Affiliations:** 1 Department of Public Health, Hyogo Medical University, Nishinomiya 663-8501, Hyogo, Japan; 2 Department of Infection Control and Prevention, Hyogo Medical University, Nishinomiya 663-8501, Hyogo, Japan; nakajima@hyo-med.ac.jp (K.N.); yamakumi@hyo-med.ac.jp (K.Y.); i-kaori@hyo-med.ac.jp (K.I.); ichiki@hyo-med.ac.jp (K.I.); taka76@hyo-med.ac.jp (T.U.); takesuey@hyo-med.ac.jp (Y.T.); 3 Department of Legal Medicine, Hyogo Medical University, Nishinomiya 663-8501, Hyogo, Japan; tk-yamamoto@hyo-med.ac.jp; 4 Department of Respiratory Medicine and Hematology, Hyogo Medical University, Nishinomiya 663-8501, Hyogo, Japan; parasol@mua.biglobe.ne.jp; 5 Department of Public Health Nursing, Mie University Graduate School of Medicine, Tsu 514-0001, Mie, Japan; aruminat@gmail.com; 6 The Research Foundation for Microbial Diseases of Osaka University, Kanonji 768-0065, Kagawa, Japan; yinai@mail.biken.or.jp; 7 Department of Microbiology, Hyogo Medical University, Nishinomiya 663-8501, Hyogo, Japan; tmokuno@hyo-med.ac.jp

**Keywords:** influenza vaccination, humoral immunity, cellular immunity, neutralizing antibody, hemagglutination inhibition, interferon-gamma (IFN-γ)

## Abstract

Assessment of the immune response to influenza vaccines should include an assessment of both humoral and cell-mediated immunity. However, there is a lack of consensus regarding the timing of immunological assessment of humoral and cell-mediated immunity after vaccination. Therefore, we investigated the timing of immunological assessments after vaccination using markers of humoral and cell-mediated immunity. In the 2018/2019 influenza season, blood was collected from 29 healthy adults before and after vaccination with a quadrivalent inactivated influenza vaccine, and we performed serial measurements of humoral immunity (hemagglutination inhibition [HAI] and neutralizing antibody [NT]) and cell-mediated immunity (interferon-gamma [IFN-γ]). The HAI and NT titers before and after vaccination were strongly correlated, but no correlation was observed between the markers of cell-mediated and humoral immunity. The geometric mean titer and geometric mean concentration of humoral and cellular immune markers increased within 2 weeks after vaccination and had already declined by 8 weeks. This study suggests that the optimal time to assess the immune response is 2 weeks after vaccination. Appropriately timed immunological assessments can help ensure that vaccination is effective.

## 1. Introduction

Influenza A/H1N1 and A/H3N2 outbreaks are common worldwide. In Japan, an outbreak of influenza A/H1N1 caused a marked increase in the incidence of influenza in the first half of the 2018/2019 season and was followed by an outbreak of influenza A/H3N2 in the second half of the season. Detection of the influenza B virus remains low [1]. During the 2018/2019 influenza season in Japan, the proportion of positive tests attributable to A/H1N1, A/H3N2, and B/Victoria lineage was 56%, 36%, and 8%, respectively [1,2]. In the United States, an outbreak of influenza A/H1N1 occurred in the initial part of the season, and an outbreak of influenza A/H3N2 occurred in February and March 2019 [3,4]. Outbreaks of influenza A/H1N1 and A/H3N2 also occurred in Europe during this period, but no influenza B outbreaks were reported [5,6].

In recent years, several studies have been conducted to evaluate the effectiveness of seasonal influenza vaccines using a test-negative design. The effectiveness of seasonal influenza vaccines depends on the antigenic match between the prevalent virus types and the antigenicity of the vaccine strains; therefore, the effectiveness of vaccination at seasonal influenza prevention must be assessed on a season-by-season basis. In the United States, the vaccine efficacy for preventing influenza virus infection overall and A/H1N1 specifically, is 29% and 44%, respectively; however, the vaccine efficacy for preventing A/H3N2 is only 9% [3,7]. In Japan, the vaccine efficacy for preventing influenza virus infection overall, and for A/H1N1, and A/H3N2 are 41.7%, 56.2%, and 34.5%, respectively [8].

The onset, type, and magnitude of seasonal influenza epidemics vary widely from year to year and are unpredictable even in regions where influenza epidemics occur annually. The timing and magnitude of an epidemic are influenced by several factors, including antigenic variation in the virus, virulence, degree of infectivity, degree of immunity of the population, and the characteristics of the population. During the coronavirus disease (COVID-19) pandemic, seasonal influenza declined globally because of the effectiveness of COVID-19 infection control measures. The basic reproduction number (R0) of influenza is approximately 1.5–2. The incubation period ranges from 1 to 4 days with an average of 2 days [9,10,11]. Thus, infection may spread rapidly in vulnerable populations during epidemics. Individuals at risk of severe disease and complications include those with specific underlying diseases, young children, and older adults [11]. In persons aged ≥65 years, influenza vaccines may be less effective than in younger persons; nevertheless, influenza vaccination decreases the risk of complications and mortality rates in older adults.

Influenza vaccines may decrease the incidence of complications and mortality rates, even if they do not prevent infection. Humoral immunity-based immunological assessment is routinely performed when evaluating vaccines, and the hemagglutination inhibition (HAI) assay is the method of evaluation most frequently used. The HAI assay is simple and can be performed on serum; therefore, it can be used to evaluate herd immunity. Evaluation of cell-mediated immunity may also be necessary to assess immunity at an individual level [12]. However, no simple method is currently available for measuring cell-mediated immunity. In a previous study, we developed a method for measuring cell-mediated immunity related to influenza vaccines [13].

Consensus regarding the timing of immunological assessment of humoral and cell-mediated immunity after vaccination has not been reached. Therefore, in this study, we investigated the optimal timing of immunological assessments after vaccination using humoral and cell-mediated immunity.

## 2. Materials and Methods

### 2.1. Study Population and Vaccine

Twenty-nine healthy adult volunteers (age: 28–57 years, 13 men and 16 women) were enrolled in this study. The participants were vaccinated against influenza between September and December 2018. A quadrivalent inactivated influenza vaccine (IIV4) (BIKEN, Osaka, Japan, Lot HA181B) was used, and a 0.5 mL dose was injected subcutaneously. Blood was collected before influenza vaccination as well as 2 weeks, 8 weeks, and 5 months after vaccination. Cell-mediated immunity (interferon-gamma [IFN-γ]) was measured before vaccination as well as 2 and 8 weeks after vaccination. Humoral immunity (HAI and neutralizing antibodies [NT]) was measured before vaccination as well as 2 weeks, 8 weeks, and 5 months after vaccination. All volunteers were vaccinated against influenza in the 2017/2018 season.

This study was approved by the Ethics Review Board of Hyogo Medical University (protocol number: 1592).

### 2.2. Antigen

Since the 2015/16 influenza season, IIV4 with four influenza virus vaccine strains, namely type A/subtype H1N1, type A/subtype H3N2, type B/Victoria lineage, and type B/Yamagata lineage, has been used for influenza vaccination in Japan. The A/H3N2 and B/Victoria lineage strains were changed during the 2017/2018 season. We used the four vaccine antigens that were used in the IIV4 vaccine in the 2018/2019 season, namely A/Singapore/GP1908/2015 IVR-180 (H1N1) pdm09, A/Singapore/INFIMH-16-0019/2016 IVR-186 (H3N2), B/Phuket/3073/2013 (B/Yamagata lineage), and B/Maryland/15/2016NYMC BX-69A (B/Victoria lineage). The vaccine antigens, including the antigens used for the IFN-γ release assay were provided by BIKEN.

### 2.3. Antibody Titration (HAI and NT)

The HAI antibody titer was measured using the vaccine strains. To remove non-specific inhibitors, each sample was treated with a receptor-destroying enzyme (RDE (II) “SEIKEN”, Denka Seiken Co., Tokyo, Japan), and diluted 1:10. Serum HAI antibody levels were measured using an influenza virus HAI assay (Denka Seiken Co., Tokyo, Japan). The final dilution ratio of samples that completely inhibited hemagglutination was regarded as the HAI antibody titer. Samples with an HAI antibody titer of ≥1:10 were evaluated as positive, and those with an HAI antibody titer of <1:10 were evaluated as negative. The HAI antibody titer was measured in a commercial laboratory (SRL Inc., Tokyo, Japan).

Serum levels of NT against the vaccine viruses were measured using micro-neutralization assays as previously described [14,15] with minor modifications. Samples with an NT titer of ≥1:10 were classified as positive, and those with an NT titer of <1:10 were classified as negative.

### 2.4. IFN-γ Assay

The IFN-γ assay was performed as previously described [13]. A total of 100 µL of heparinized whole blood and each influenza antigen (HA titer, 10 µg/mL) diluted in Roswell Park Memorial Institute (RPMI) 1640 medium were added to 96-well microtiter plates to a final volume of 200 µL/well for incubation. The assay plates were incubated at 36.5 °C in 5% CO_2_. Co-cultivations were conducted within 1 h of drawing the blood samples. The culture supernatants (100 μL) were collected after 48 h of cultivation and stored at −80 °C. IFN-γ was measured using the supernatants. The IFN-γ concentration was quantified using an enzyme-linked immunosorbent assay (IFN-γ Human Elisa Kit; eBioscience, San Diego, CA, USA) according to the manufacturer’s instructions.

The medium rather than the influenza antigen was added to the blood to serve as the negative control. The amount of IFN-γ released in the negative control wells in all experiments was <4 pg/mL.

In our previous study, none of the participants with influenza after vaccination had a greater than 1.5-fold increase in IFN-γ concentration after vaccination; therefore, a ≥1.5-fold increase in IFN-γ was regarded as positive (protective against infection) [13]. In this study, the four antigens (A/H1N1, A/H3N2, B/Yamagata lineage, and B/Victoria lineage) were quantitatively evaluated using geometric mean concentration (GMC) and geometric mean concentration ratio (GMCR).

### 2.5. Changes in Antibodies after Influenza Vaccination

The European Medicines Agency guidelines [16] specify that evaluation of the immune response to influenza vaccines in individuals aged 18 to 59 years should include the HAI antibody titer before and after vaccination and that at least one of the following three criteria should be met for vaccine effectiveness:

1. Criterion 1: ≥70% of individuals have an HAI antibody titer ≥ 1:40 after vaccination;
2. Criterion 2: ≥40% of individuals with a negative HAI antibody titer pre-vaccination have an HAI antibody titer ≥ 1:40 post-vaccination or a ≥4-fold rise in HAI antibody titer in those with detectable HAI antibodies pre-vaccination;
3. Criterion 3: A GMCR > 2.5.

There are no specific guidelines for evaluating the immune response using the NT antibody titer. According to a previous study, a serum HAI antibody titer of 1:40 generally corresponds with an NT titer of approximately 1:160 [17]. Therefore, in this study, the criteria were evaluated as follows:

1. Criterion 1: ≥70% of participants with an HAI titer ≥ 1:40 or an NT titer ≥ 1:160 after vaccination.
2. Criterion 2: ≥40% of participants with a change in HAI titer from negative pre-vaccination to ≥1:40 post-vaccination or a ≥4-fold rise in HAI titer; or a change in NT titer from negative pre-vaccination to ≥1:160 post-vaccination or a ≥4-fold rise in NT titer.
3. Criterion 3: Proportion of participants with a GMCR > 2.5.

### 2.6. Statistical Analyses

Spearman’s rank correlation coefficient (ρ) was employed to assess the correlation between the results of the HAI and NT assays with significance level of *p* < 0.01. All statistical analyses were conducted using SPSS version 29 (IBM Corp., Armonk, NY, USA).

## 3. Results

### 3.1. Changes in Antibody Titers after Influenza Vaccination

#### 3.1.1. Criterion 1: Proportion of Participants with an HAI Titer ≥ 1:40 or NT Titer ≥ 1:160

None of the four vaccine antigens satisfied the condition of ≥70% of participants having an HAI titer ≥ 1:40 after vaccination. Using the NT method, ≥70% of participants had an NT titer ≥ 1:160 for H1N1 (83% after 2 weeks, 72% after 8 weeks, and 72% after 5 months), H3N2 (97% after 2 weeks, 93% after 8 weeks, and 90% after 5 months), and B/Yamagata lineage (76% after 2 weeks) (Table 1).

#### 3.1.2. Criterion 2: Proportion of Participants with a ≥4-Fold Increase in HAI or NT Antibody Titer or Change from Negative to ≥1:40 or ≥1:160 for HAI and NT Titer, Respectively

Two weeks after vaccination, the proportion of participants with a change in HAI titer from negative to >1:40 or a more than four-fold increase in HAI titer was 3.4% (1/29), 6.9% (2/29), 0% (0/29), and 3.4% (1/29) for A/H1N1, A/H3N2, B/Yamagata lineage, and B/Victoria lineage, respectively.

Two weeks after vaccination, the proportion of participants with a change in NT titer from negative to >1:160 or a more than four-fold increase was 0% (0/29), 3% (1/29), 0% (0/29), and 3% (1/29) for A/H1N1, A/H3N2, B/Yamagata lineage, and B/Victoria lineage, respectively. None of the four vaccine antigens met Criterion 2 (Table 2).

#### 3.1.3. Criterion 3: Geometric Mean Titer (GMT) of HAI and NT Pre- and Post-Vaccination and Geometric Mean Concentration Ratio (GMCR)

Two weeks after vaccination, the GMCR for HAI antibodies was 1.4, 1.3, 1.3, and 1.2 for A/H1N1, A/H3N2, B/Yamagata lineage, and B/Victoria lineage, respectively. The GMCR for NT antibodies was 1.7, 2.0, 1.4, and 1.6 for A/H1N1, A/H3N2, B/Yamagata lineage, and B/Victoria lineage, respectively. None of the HAI and NT GMCR values were ≥2.5 for any of the four influenza virus antigens (Table 3).

### 3.2. Cell-Mediated Immunity (IFN-γ) before and after Influenza Vaccination

IFN-γ was measured with respect to four antigens (A/H1N1, A/H3N2, B/Yamagata lineage, and B/Victoria lineage) pre-vaccination as well as 2 and 8 weeks post-vaccination. The four influenza virus antigens were quantitatively evaluated using GMC and GMCR. The GMC and GMCR of IFN-γ were increased 2 weeks post-vaccination but had decreased by 8 weeks post-vaccination (Table 4).

The number (%) of participants with an IFN-γ GMCR ≥ 1.5, 2–3 weeks after vaccination was 12 (41%), 11 (38%), 8 (28%), and 11 (38%), for A/H1N1, A/H3N2, B/Yamagata lineage, and B/Victoria lineage, respectively. The number of participants with an IFN-γ GMCR ≥ 1.5, 8 weeks after vaccination was 7, 5, 9, and 7 for A/H1N1, A/H3N2, B/Yamagata lineage, and B/Victoria lineage, respectively (Table 5). The number of participants with a GMCR ≥ 1.5 was lower at 8 weeks than at 2 weeks after vaccination for all antigens other than B/Yamagata lineage (Table 5).

### 3.3. Relationship between HAI and NT before and after Vaccination

The correlation between HAI and NT before vaccination and 2 weeks, 8 weeks, and 5 months post-vaccination were calculated for the four influenza vaccine antigens (A/H1N1, A/H3N2, B/Yamagata lineage, and B/Victoria lineage) using Spearman’s correlation coefficient (Table 6). The correlation coefficients pre-vaccination were 0.85, 0.80, 0.73, and 0.82 for A/H1N1, A/H3N2, B/Yamagata lineage, and B/Victoria lineage, respectively. The correlation coefficients 2 weeks post-vaccination were 0.92, 0.82, 0.86, and 0.90 for A/H1N1, A/H3N2, B/Yamagata lineage, and B/Victoria lineage, respectively. The correlation coefficients 8 weeks post-vaccination were 0.87, 0.90, 0.93, and 0.91 for A/H1N1, A/H3N2, B/Yamagata lineage, and B/Victoria lineage, respectively. The correlation coefficients 5 months post-vaccination were 0.90, 0.86, 0.87, and 0.73 for A/H1N1, A/H3N2, B/Yamagata lineage, and B/Victoria lineage, respectively (Table 6).

The HAI and NT titers showed statistically significant correlations (*p* < 0.01) at all time points and for all antigens. The strongest correlation was found for the B/Yamagata lineage 8 weeks post-vaccination of (ρ = 0.93), and the weakest correlation was found for the B/Victoria lineage 5 months post-vaccination (ρ = 0.72). These results suggest that HAI and NT respond similarly to influenza vaccination. However, there was no significant correlation between cell-mediated immunity (IFN-γ) and antibodies (HAI and NT) before vaccination.

## 4. Discussion

In this study, we investigated changes in humoral immunity (HAI and NT) and cell-mediated immunity (IFN-γ) at 2 and 8 weeks after influenza vaccination. Compared with the pre-vaccination values, the GMT and GMCR of HAI and NT were increased 2 weeks after vaccination but had decreased by 8 weeks after vaccination. Similarly, the IFN-γ concentrations were increased 2 weeks after vaccination but had decreased by 8 weeks after vaccination. Notably, the IFN-γ concentrations had returned to pre-vaccination levels by 8 weeks after vaccination. Therefore, humoral and cell-mediated immunity-based immunological assessments should be performed 2 weeks after vaccination rather than 8 weeks after vaccination.

In a National Epidemiological Surveillance of Vaccine Preventable Diseases survey, pre-vaccination HAI antibody titers were measured in 6569 serum samples collected in Japan [1,18]. The proportion of individuals with an HAI antibody titer of ≥1:40 before vaccination differed according to age and influenza antigen type. The proportion of individuals with an HAI titer of ≥1:40 before vaccination was high (≥60%) in those aged 10 to 24 years, 5 to 24 years, and 20 to 34 years for A/H1N1, A/H3N2, and B/Yamagata lineage, respectively, but it was low (<60%) for B/Victoria lineage in all age groups [1,18]. In this study, the antibody titer before vaccination was generally low, which was possibly because 15 of the 29 participants were aged 40–49 years. A previous study found that the risk of influenza A/H3N2 was halved when the HAI titer increased four-fold or more after vaccination, but the study did not evaluate the predictive power of the HAI response [19]. In this study, only 2 of the 29 participants had a greater than four-fold increase in the HAI titer for A/N3H2. When a virus drifts antigenically from a circulating vaccine virus, the efficacy of the vaccine may be reduced even if the antibody titer for the vaccine virus is high. Furthermore, host factors such as age and underlying diseases may influence the immune response to vaccination [11].

Viral virus-neutralizing titers are an important indicator of protection. However, anti-influenza virus activity is usually measured based on the HAI titer. This assay assesses the inhibition of viral particles bound to sialic acid. In contrast, the NT assay assesses the inhibition of viral attachment and entry into cells and the release of progeny viral particles [14]. NT is considered a more important protection antibody than HAI, but the relationship between NT activity and level of protection has not been assessed in detail. NT is not commonly used in virological studies because the test is time-consuming and does not allow for multiple specimens to be processed simultaneously. The NT assay is more sensitive than the HAI assay for measuring humoral immunity against influenza viruses [17]. However, the HAI method is used more frequently than the NT method because it is simpler. An HAI antibody titer of ≥1:40 is used as a gold standard for protective levels of antibodies and reflects the level at which approximately 50% of individuals are protected from infection [20]. According to a previous study, a serum HAI antibody titer of 1:40 is generally associated with a neutralizing antibody titer of approximately 1:160 [17]. Therefore, the neutralizing antibody titer required for protection was assumed to be 1:160 in this study, and the HAI and NT titers were compared. The HAI titer and the NT titer have previously been shown to be significantly correlated after administering two doses of vaccine [21]. In this study, serial immunological assessments after vaccination showed a strong and significant correlation between the HAI and NT titers before vaccination and 2 weeks, 8 weeks, and 5 months after vaccination.

Cell-mediated immunity helps to eliminate viruses and establish immune memory, contributing to protection against infection, a decrease in the viral load, and recovery. However, no methods have been established to measure protection provided by cell-mediated immunity [11,22] because no simple methods are available to quantify cell-mediated immunity. Thus, a simple method was developed to quantify cell-mediated immunity by reacting whole blood with antigens and using IFN-γ as an indicator of the cellular immune response [13]. Individuals in whom influenza vaccination does not induce IFN-γ are more susceptible to influenza, suggesting that the induction of IFN-γ is related to protection against infection [13]. An alternative method of measuring cell-mediated immunity is isolating and reacting the cells with antigens. A comparison of the two methods revealed a significant correlation [23]. Therefore, we measured cell-mediated immunity using a whole blood method, which is simple and straightforward.

The US Centers for Disease Control and Prevention (CDC) recommends that children receive two doses of influenza vaccine administered between the ages of 6 months and 8 years if the indications are met [24]. Few studies have assessed the effect of two doses of vaccine on cell-mediated immunity. One study showed that 50% of individuals without protective levels of cell-mediated immunity after one dose of vaccine developed protective levels after a second dose of vaccine, but that cell-mediated immunity was not enhanced by a second dose of vaccine in individuals in whom one dose of vaccine induced cell-mediated immunity [21]. In addition to IGN-γ, granzyme B, which plays an important role in cytotoxic T lymphocytes (CTL), may play an important role in cell-mediated immunity. IFN-γ and granzyme B are both stimulated by influenza vaccination, and IFN-γ and granzyme B levels are correlated [24,25]. This suggests that monitoring changes in IFN-γ could be useful as an indicator of CTL activity. Furthermore, it may be useful to evaluate cell-mediated immunity in addition to humoral immunity when assessing susceptibility to infection [11]. The results of this study suggest that the optimal time for measuring vaccine-induced cell-mediated immunity is 2 weeks after vaccination.

New more effective influenza vaccines are being developed. In the United States, the quadrivalent live-attenuated influenza vaccine (LAIV4; Flumist) was approved for use in healthy, non-pregnant individuals aged 2–49 years in 2003 [26]. LAIV4 was approved in Japan in 2023. This vaccine reduces the risk of influenza by 80% in children, even in seasons when the antigenicity of the epidemic virus strain and the vaccine strain do not match [26]. Data from healthy adults have shown that IIV4 is more effective than LAIV4 [27]; however, the results vary according to the prevalent influenza virus type, country, and region.

In Japan, the incidence of influenza was low during the 2020/2021 and 2021/2022 seasons, whereas influenza epidemics occurred in the 2022/2023 and 2023/2024 seasons albeit at a lower level than that prior to the COVID-19 pandemic [28]. During the 2023/2024 season, there were concurrent epidemics of COVID-19 and influenza. The incidence of influenza may return to pre-COVID-19 levels during the 2024/2025 season. It is likely that annual seasonal influenza epidemics will continue to occur in the future. As vaccine antigens are updated annually, annual vaccination is recommended for all persons [29].

This study has some limitations. The sample size was small, and it was not possible to perform analyses according to age. The antibody prevalence before vaccination varies according to age; hence, future studies should evaluate the antibody prevalence before vaccination according to age. Furthermore, none of the participants had influenza; therefore, infection-related changes in humoral and cell-mediated immunity could not be evaluated. Changes in humoral and cell-mediated immunity need to be assessed in infected individuals so that differences in vaccine-related immunity can be assessed.

## 5. Conclusions

Late assessment of immunological responses to influenza vaccination could lead to the conclusion that immunological response to vaccination is inadequate. Even when the levels of markers of humoral (HAI and NT) and cell-mediated (IFN-γ) immunity decrease, immune memory may remain. To our knowledge, this study is the first to assess the timing of changes in cell-mediated immunity following influenza vaccination. It shows that an immunological assessment of vaccines based on humoral or cell-mediated immunity should ideally be performed 2 weeks after vaccination. Adequate assessment of the humoral and cellular immune response might reduce influenza-related morbidity and mortality and could also contribute to the development of more effective influenza vaccines and methods of vaccination.

## Figures and Tables

**Table 1:** Proportion of participants with an HAI titer ≥ 1:40 and an NT titer ≥ 1:160 (N = 29).

Influenza Vaccine Antigen	Proportion of Participants with HAI ≥ 1:40				Proportion of Participants with NT ≥ 1:160			
	Pre	2 wk	8 wk	5 mo	Pre	2 wk	8 wk	5 mo
A/H1N1	31% (9/29)	48% (14/29)	41% (12/29)	34% (10/29)	69% (20/29)	83% (24/29)	72% (21/29)	72% (21/29)
A/H3N2	38% (11/29)	52% (15/29)	38% (11/29)	34% (10/29)	79% (23/29)	97% (28/29)	93% (27/29)	90% (26/29)
B/Yamagata lineage	52% (15/29)	59% (17/29)	59% (17/29)	55% (16/29)	62% (18/29)	76% (22/29)	62% (18/29)	66% (16/29)
B/Victoria lineage	38% (11/29)	38% (11/29)	38% (11/29)	34% (10/29)	45% (13/29)	59% (17/29)	45% (13/29)	45% (13/29)

HAI, hemagglutination inhibition; NT, neutralizing antibody; Pre, pre-vaccination; wk, weeks post-vaccination; mo, months post-vaccination.

**Table 2:** Proportion of participants with a ≥4-fold rise in HAI titer or NT titer or change from negative to ≥1:40 or ≥1:160 for HAI and NT titer, respectively (N = 29).

Influenza Vaccine Antigen	HAI (≥4-Fold Rise or Change from Negative to ≥1:40)			NT (≥4-Fold Rise or Change from Negative to ≥1:160)		
	2 Weeks	8 Weeks	5 Months	2 Weeks	8 Weeks	5 Months
A/H1N1	3% (1/29)	3% (1/29)	0% (0/29)	0% (0/29)	0% (0/29)	0% (0/29)
A/H3N2	7% (2/29)	0% (0/29)	0% (0/29)	3% (1/29)	3% (1/29)	3% (1/29)
B/Yamagata lineage	0% (0/29)	0% (0/29)	0% (0/29)	0% (0/29)	0% (0/29)	0% (0/29)
B/Victoria lineage	3% (1/29)	3% (1/29)	0% (0/29)	3% (1/29)	0% (0/29)	0% (0/29)

HAI, hemagglutination inhibition; NT, neutralizing antibody; weeks, weeks post-vaccination; months, months post-vaccination.

**Table 3:** Geometric mean titers (GMCs) pre- and post-vaccination and geometric mean concentration ratio (GMCR) (N = 29).

Influenza Vaccine Antigen	HAI GMT Pre- and Post-Vaccination (GMCR)				NT GMT Pre- and Post-Vaccination (GMCR)			
	Pre	2 Weeks	8 Weeks	5 Months	Pre	2 Weeks	8 Weeks	5 Months
A/H1N1	16.5	23.6 (1.4)	21.1 (1.3)	16.5 (1)	141	246.6 (1.7)	176.4 (1.3)	166.3 (1.2)
A/H3N2	16.9	23.6 (1.3)	19.7 (1.1)	18.1 (1.1)	230.6	461.3 (2.0)	344.9 (1.5)	317.6 (1.4)
B/Yamagata lineage	22	29.5 (1.3)	25.2 (1.1)	26.2 (1.2)	123.3	172.5 (1.4)	115.3 (0.94)	112.3 (0.91)
B/Victoria lineage	20.6	24.7 (1.2)	21.1 (1.0)	21.3 (1.0)	100.8	161.3 (1.6)	117.9 (1.2)	116.5 (1.2)

HAI, hemagglutination inhibition; NT, neutralizing antibody; GMT, geometric mean titer; GMCR, geometric mean concentration ratio; pre, pre-vaccination; weeks, weeks post-vaccination; months, months post-vaccination.

**Table 4:** Geometric mean concentration (GMC) and geometric mean concentration ratio (GMCR) on stimulation of whole blood with vaccine antigens (N = 29).

Influenza Vaccine Antigen	GMC (GMCR)		
	Pre	2 Weeks	8 Weeks
A/H1N1	85.3	103.1 (1.2)	71.9 (0.8)
A/H3N2	62.6	82.4 (1.3)	58.8 (0.9)
B/Yamagata lineage	92.9	110.2 (1.2)	83.1 (0.9)
B/Victoria lineage	69.6	91.2 (1.3)	70.8 (1.0)

GMC, geometric mean concentration; GMCR, geometric mean concentration ratio; pre, pre-vaccination; weeks, weeks post-vaccination.

**Table 5:** Proportion of participants with an IFN-γ geometric mean concentration ratio (GMCR) ≥ 1.5, at 2 and 8 weeks post-vaccination (N = 29).

Influenza Vaccine Antigen	GMCR ≥ 1.5	
	2 Weeks Post-Vaccination	8 Weeks Post-Vaccination
H1N1	41% (12/29)	24% (7/29)
H3N2	38% (11/29)	17% (5/29)
B/Yamagata lineage	28% (8/29)	31% (9/29)
B/Victoria lineage	38% (11/29)	24% (7/29)

GMCR, geometric mean concentration ratio.

**Table 6:** Spearman’s correlation coefficients for assessing the relationship between HAI and NT titers before and after vaccination (N = 29).

Influenza Vaccine Antigen	Correlation between HAI and NT Titers (Spearman’s ρ)			
	Pre-	2 Weeks	8 Weeks	5 Months
A/H1N1	0.85 **	0.80 **	0.73 **	0.82 **
A/H3N2	0.92 **	0.82 **	0.86 **	0.90 **
B/Yamagata lineage	0.87 **	0.90 **	0.93 **	0.91 **
B/Victoria lineage	0.90 **	0.86 **	0.87 **	0.72 **

HAI, hemagglutination inhibition; NT, neutralizing antibody; pre, pre-vaccination; weeks, weeks post-vaccination; months, months post-vaccination. ** *p* < 0.01.

## Data Availability

The raw data supporting the conclusions of this article will be made available by the authors on request.

## References

[1] National Institute of Infectious Diseases (NIID) (2019). Influenza 2018/2019 Season. IASR.

[2] National Institute of Infectious Diseases (NIID) Isolation/Detection of Influenza Viruses during the 2018/2019 Influenza Season. Table 2. https://www.niid.go.jp/niid/images/iasr/2019/11/477tt02.gif.

[3] US Centers for Disease Control and Prevention (CDC) US Flu VE Data for 2018–2019. https://archive.cdc.gov/www_cdc_gov/flu/vaccines-work/2018-2019.html.

[4] Flannery B., Garten Kondor R.J.G., Chung J.R., Gaglani M., Reis M., Zimmerman R.K., Nowalk M.P., Jackson M.L., Jackson L.A., Monto A.S. (2020). Spread of antigenically drifted influenza A(H3N2) viruses and vaccine effectiveness in the United States during the 2018–2019 season. J. Infect. Dis..

[5] European Centre for Disease Prevention and Control (ECDC), World Health Organization Regional Office for Europe Flu News Europe. Summary Week 3/2019 (14–20 January 2019). https://flunewseurope.org/Archives.

[6] Kissling E., Rose A., Emborg H.D., Gherasim A., Pebody R., Pozo F., Trebbien R., Mazagatos C., Whitaker H., Valenciano M. (2019). Interim 2018/19 influenza vaccine effectiveness: Six European studies, October 2018 to January 2019. Euro. Surveill..

[7] Doyle J.D., Chung J.R., Kim S.S., Gaglani M., Raiyani C., Zimmerman R.K., Nowalk M.P., Jackson M.L., Jackson L.A., Monto A.S. (2019). Interim estimates of 2018–2019 seasonal influenza vaccine effectiveness–United States, February 2019. MMWR Morb. Mortal. Wkly. Rep..

[8] Ando S. (2020). Estimation of the effectiveness of quadrivalent influenza vaccines by distinguishing between influenza A (H1N1) pdm09 and influenza A (H3N2) using rapid influenza diagnostic tests during the 2018–2019 season. Intern. Med..

[9] Fraser C., Donnelly C.A., Cauchemez S., Hanage W.P., Van Kerkhove M.D., Hollingsworth T.D., Griffin J., Baggaley R.F., Jenkins H.E., Lyons E.J. (2009). Pandemic potential of a strain of influenza A (H1N1): Early findings. Science.

[10] Truscott J., Fraser C., Hinsley W., Cauchemez S., Donnelly C., Ghani A., Ferguson N., Meeyai A. (2009). Quantifying the transmissibility of human influenza and its seasonal variation in temperate regions. PLoS Curr..

[11] Bresee J.S., Fly A.M., Sambhara S., Cox N.J., Plotkin S.A., Orenstein W.A., Offit P.A., Edwards K.M. (2018). Inactivated influenza vaccines. Plotkin’s Vaccines.

[12] Hamborsky J., Kroger A., Wolfe S., Centers for Disease Control and Prevention (CDC) (2015). Influenza. Epidemiology and Prevention of Vaccine-Preventable Diseases.

[13] Otani N., Shima M., Ueda T., Ichiki K., Nakajima K., Takesue Y., Okuno T. (2016). Evaluation of influenza vaccine-immunogenicity in cell-mediated immunity. Cell Immunol..

[14] Okuno Y., Tanaka K., Baba K., Maeda A., Kunita N., Ueda S. (1990). Rapid focus reduction neutralization test of influenza A and B viruses in microtiter system. J. Clin. Microbiol..

[15] Onodera H., Urayama T., Hirota K., Maeda K., Kubota-Koketsu R., Takahashi K., Hagiwara K., Okuno Y., Ikuta K., Yunoki M. (2017). Neutralizing activities against seasonal influenza viruses in human intravenous immunoglobulin. Biologics.

[16] The European Agency for the Evaluation of Medicinal Products (EMEA), Committee for Proprietary Medicinal Products Note for Guidance on Harmonization of Requirements for Influenza Vaccines. CPMP/BWP/214/96. https://www.ema.europa.eu/en/documents/scientific-guideline/note-guidance-harmonisation-requirements-influenza-vaccines_en.pdf.

[17] Verschoor C.P., Singh P., Russell M.L., Bowdish D.M., Brewer A., Cyr L., Ward B.J., Loeb M. (2015). Microneutralization assay titres correlate with protection against seasonal influenza H1N1 and H3N2 in children. PLoS ONE.

[18] National Institute of Infectious Diseases Influenza Antibody Status. 2018 (Japanese). https://www.niid.go.jp/niid/ja/flu-m/253-idsc/yosoku/sokuhou/8504-flu-yosoku-rapid2018-1.html.

[19] Benoit A., Beran J., Devaster J.M., Esen M., Launay O., Leroux-Roels G., McElhaney J.E., Oostvogels L., van Essen G.A., Gaglani M. (2015). Hemagglutination inhibition antibody titers as a correlate of protection against seasonal A/H3N2 influenza disease. Open Forum. Infect. Dis..

[20] Hobson D., Curry R.L., Beare A.S., Ward-Gardner A. (1972). The role of serum haemagglutination-inhibiting antibody in protection against challenge infection with influenza A2 and B viruses. J. Hyg..

[21] Otani N., Shima M., Ueda T., Ichiki K., Nakajima K., Takesue Y., Honjo K., Yoshida N., Kawata S., Okuno T. (2018). Relationship between the frequency of influenza vaccination and cell-mediated immunity. J. Immunol. Methods.

[22] Wilkinson T.M., Li C.K., Chui C.S., Huang A.K., Perkins M., Liebner J.C., Lambkin-Williams R., Gilbert A., Oxford J., Nicholas B. (2012). Preexisting influenza-specific CD4+ T cells correlate with disease protection against influenza challenge in humans. Nat. Med..

[23] Otani N., Shima M., Ueda T., Ichiki K., Nakajima K., Takesue Y., Okuno T. (2019). Evaluation of influenza vaccine-induced cell-mediated immunity: Comparison between methods using peripheral blood mononuclear cells and whole blood. Microbiol. Immunol..

[24] US Centers for Disease Control and Prevention Summary: Prevention and Control of Seasonal Influenza with Vaccines: Recommendations of the Advisory Committee on Immunization Practices (ACIP)—United States, 2023–2024. https://www.cdc.gov/flu/professionals/acip/summary/summary-recommendations.htm#concurrent.

[25] Otani N., Nakajima K., Ishikawa K., Ichiki K., Ueda T., Takesue Y., Yamamoto T., Tanimura S., Shima M., Okuno T. (2021). Changes in cell-mediated immunity (IFN-γ and granzyme B) following influenza vaccination. Viruses.

[26] Patel M.M., Grohskopf L.A., Sambhara S., Belser J.A., Katz J.M., Fly A.M., Prenstein W.A., Offit P.A., Edvards K.M., Plotkin S.A. (2023). Inactivated and Recombinant Influenza Vaccines. Plotkin’s Vaccines.

[27] Monto A.S., Ohmit S.E., Petrie J.G., Johnson E., Truscon R., Teich E., Rotthoff J., Boulton M., Victor J.C. (2009). Comparative efficacy of inactivated and live attenuated influenza vaccines. N. Engl. J. Med..

[28] National Institute of Infectious Diseases Influenza Cases Reported per Sentinel Weekly. https://www.niid.go.jp/niid/ja/10/2096-weeklygraph/1644-01flu.html.

[29] Grohskopf L.A., Alyanak E., Ferdinands J.M., Broder K.R., Blanton L.H., Talbot H.K., Fry A.M. (2021). Prevention and control of seasonal influenza with vaccines: Recommendations of the Advisory Committee on Immunization Practices, United States, 2021–2022 influenza season. MMWR Recomm. Rep..

